# Perfusion Imaging in Pusher Syndrome to Investigate the Neural Substrates Involved in Controlling Upright Body Position

**DOI:** 10.1371/journal.pone.0005737

**Published:** 2009-05-29

**Authors:** Luca Francesco Ticini, Uwe Klose, Thomas Nägele, Hans-Otto Karnath

**Affiliations:** 1 Section of Neuropsychology, Center of Neurology, Hertie-Institute for Clinical Brain Research, University of Tübingen, Tübingen, Germany; 2 Department of Diagnostic and Interventional Neuroradiology, University Hospital of Tübingen, Tübingen, Germany; Julius-Maximilians-Universität Würzburg, Germany

## Abstract

Brain damage may induce a dysfunction of upright body position termed “pusher syndrome”. Patients with such disorder suffer from an alteration of their sense of body verticality. They experience their body as oriented upright when actually tilted nearly 20 degrees to the ipsilesional side. Pusher syndrome typically is associated with posterior thalamic stroke; less frequently with extra-thalamic lesions. This argued for a fundamental role of these structures in our control of upright body posture. Here we investigated whether such patients may show additional functional or metabolic abnormalities outside the areas of brain lesion. We investigated 19 stroke patients with thalamic or with extra-thalamic lesions showing versus not showing misperception of body orientation. We measured fluid-attenuated inversion-recovery (FLAIR) imaging, diffusion-weighted imaging (DWI), and perfusion-weighted imaging (PWI). This allowed us to determine the structural damage as well as to identify the malperfused but structural intact tissue. Pusher patients with thalamic lesions did not show dysfunctional brain areas in addition to the ones found to be structurally damaged. In the pusher patients with extra-thalamic lesions, the thalamus was neither structurally damaged nor malperfused. Rather, these patients showed small regions of abnormal perfusion in the structurally intact inferior frontal gyrus, middle temporal gyrus, inferior parietal lobule, and parietal white matter. The results indicate that these extra-thalamic brain areas contribute to the network controlling upright body posture. The data also suggest that damage of the neural tissue in the posterior thalamus itself rather than additional malperfusion in distant cortical areas is associated with pusher syndrome. Hence, it seems as if the normal functioning of both extra-thalamic as well as posterior thalamic structures is integral to perceiving gravity and controlling upright body orientation in humans.

## Introduction

Human species is the only obligate biped among primates. Our brain thus has become remarkably efficient in stabilizing the upright body position in space. The perception of our body orientation is achieved by the convergence of inputs from multiple sources, including vestibular, visual, and somatosensory information [1]. When these sensory channels work properly, their inputs and their integration indicate verticality in a congruent manner. Damage to this system causes diverse disorders of posture and of balance control [2]–[7]. Among them, a very intriguing and severe disorder of upright body position is the “pusher syndrome” (for review ref. [8]).

Patients with pusher syndrome suffer from an alteration of their sense of body verticality [9]. They experience their body as oriented upright (subjective postural vertical, SPV) when actually tilted in the coronal (roll) plane nearly 20 degrees towards the side of the lesion [9]. The patients resist any attempt to correct passively the tilted body posture towards earth vertical upright orientation and use the non-paretic arm and/or leg to actively push towards the paralyzed side [10]. In contrast to their disturbed perception of upright body posture, their perception of the visual vertical (subjective visual vertical, SVV) meadiated by visual and vestibular input is largely preserved [9], [11]. This dissociation supported the assumption of a neural pathway in humans for sensing the orientation of gravity and controlling upright body posture, separate from the well-known visual-vestibular system for perceiving the orientation of the visual world [9], [12]–[14].

Pusher syndrome is typically associated with unilateral lesions of the posterior thalamus [13], [15], while cortical strokes sparing the thalamus [16] or non-stroke neurological aetiologies [17] are rather infrequent. These findings argued for a fundamental role of the posterior thalamus in our control of upright body posture. However, it is not yet well understood whether this disorder of upright body posture associated with thalamic strokes might also be explained by the dysfunction of cortical areas rather than by the neuronal loss in the thalamus itself. In fact, by using positron emission tomography (PET), thalamic infarctions [18]–[20] and thalamotomy [19] have been shown to induce depressed levels of metabolic activity in the cerebral hemispheres. Thus, it is possible that the thalamic lesions of pusher patients indeed cause functional or metabolic abnormalities in cortical areas via diaschisis [19], [21] or through vascular dysfunction, and that these (distant) functional abnormalities cause the patients' misperception of body orientation.

Vice versa it is possible that the rather few extra-thalamic strokes that induce pusher syndrome, i.e. the small areas within the posterior insula, superior temporal gyrus, postcentral gyrus, and inferior parietal lobule [16], might induce dysfunction through malperfusion in distant thalamic or other structurally intact neural structures.

Our aim thus was to investigate, by means of perfusion-weighted imaging (PWI), the functioning of the structurally intact cortical tissue in patients with thalamic and with extra-thalamic strokes showing versus not showing pusher syndrome. While diffusion-weighted (DWI) and fluid-attenuated inversion-recovery (FLAIR) imaging reveal information about irreversibly damaged neural tissue, PWI measures the amount and latency of blood flow reaching different regions of the brain. PWI thus allows the identification of structurally intact but abnormally perfused brain tissue; i.e. zones that are receiving enough blood supply to remain structurally intact but not enough to function normally.

## Materials and Methods

### Subjects

Nineteen patients with first-ever stroke centering either on the thalamus (n = 11) or sparing the thalamus (n = 8) consecutively admitted to the Centre of Neurology in Tübingen were included. Four patients from the latter group without thalamic involvement have also been subjects in a previous study [16] that investigated the structural aspects of brain lesions. Since stenoses are known to produce false-positive depictions of perfusion deficits, especially in time-to-peak perfusion images [22], we excluded those patients with a haemodynamically relevant extracranial stenosis in the internal carotid arteries, i.e. ≥70%, demonstrated by Doppler sonography. The number of potential participants further had to be limited with respect to proper kidney functions due to the use of contrast agent. The patients were divided into two groups with and without pusher syndrome (cf. Table 1) according to standardised testing for pusher syndrome (see below). All patients gave their written informed consent to participate in the study which has been performed in accordance with the ethical standards laid down in the 1964 Declaration of Helsinki.

**Table 1:** Demographic and clinical data of the patients with and without pusher syndrome.

		Thalamic brain lesion		Extra-thalamic brain lesion	
		Pusher syndrome	No pusher syndrome	Pusher syndrome	No pusher syndrome
Number		5	6	4	4
Sex		3f, 2m	2f, 4m	4m	2f, 2m
Age (yr)	Mean (SD)	67.8 (6.1)	56.5 (9.6)	64.5 (16.6)	64.7 (13.8)
Etiology		0 Infarct	4 Infarct	4 Infarct	4 Infarct
		5 Hemorrhage	2 Hemorrhage		
Lesion volume (% of RH)	Mean (SD)	4.8 (2.5)	2.2 (3.5)	15.9 (4.0)	8.3 (3.1)
Lesion side		2 RBD / 3 LBD	5 RBD / 1 LBD	4 RBD	4 RBD
Paresis of contralesional side	% present	100	66.6	100	100
Arm	Median (range)	2 (0–3.5)	4 (1–5)	1.1 (0–2.5)	3.5 (3–4)
Leg	Median (range)	3 (2.5–4)	5 (2–5)	2.6 (2–3)	3.5 (3–4)
Visual field deficit	% present	0	16.6	0	0
Spatial neglect (total number/max.)	LBD	0/2*	0/1	0/0	0/0
	RBD	2/2	0/5	2/4	2/4
Aphasia (total number/max.)	LBD	3/3	0/1	0/0	0/0
	RBD	0/2	0/5	0/4	0/4
SCP posture					
Sitting	Median (range)	1 (0–1)	0	1 (0.75–1)	0
Standing	Median (range)	1	0 (0–0.25)	1 (0.75–1)	0
SCP extension					
Sitting	Median (range)	1 (0.5–1)	0	0.5 (0.5–1)	0
Standing	Median (range)	1	0	0.75 (0.5–1)	0
SCP resistance					
Sitting	Median (range)	1	0	1	0
Standing	Median (range)	1	0	1	0

f, female; m, male; *, one patient could not formally be tested for spatial neglect; RH, right hemisphere; LBD, left brain damage; RBD, right brain damage; SCP, Scale for Contraversive Pushing [9], [23].

### Clinical investigation

Pusher syndrome was diagnosed using the standardised Scale for Contraversive Pushing (SCP) [9], [23] at the same day of MR acquisition. The SCP assesses 1) symmetry of spontaneous posture, 2) the use of the non-paretic arm or leg to increase pushing force by abduction and extension of extremities, and 3) resistance to passive correction of posture. These variables are determined both when patients were sitting (feet with ground contact) and standing. In patients with pusher behavior, all three criteria had to be present and the patients had to show at least a total score of 1 (max. = 2, sitting plus standing) with respect to their spontaneous posture, at least a score of 1 (max. = 2, sitting plus standing) concerning the use of the non-paretic arm and/or leg to increase pushing force by abduction and extension, and had to show resistance to passive correction of posture. Details of the SCP assessment are presented in Table 1. The degree of paresis of the upper and lower limbs was scored with the usual clinical ordinal scale, where ‘0’ stands for no trace of movement and ‘5’ for normal movement. Spatial neglect was diagnosed when the patient exhibited the typical clinical behaviour, such as spontaneous eye and head orientation towards the right [24]. In addition, all patients were further assessed with the following three clinical tests: the ‘Letter cancellation’ task [25], the ‘Bells test’ [26], and a copying task [27]. Neglect patients had to fulfill the criterion for spatial neglect in at least two of these tests. Full details about the test procedure and criteria are described elsewhere [24]. Aphasia was assessed conducting a bedside examination that evaluated spontaneous speech, auditory and reading comprehension, picture naming, reading, and oral repetition. Visual field defects were assessed using standardized neurological examination.

### MR imaging and analysis

For the depiction of structurally lesioned brain tissue we used diffusion-weighted imaging (DWI) and T2-weighted fluid-attenuated inversion-recovery (FLAIR) imaging. DWI is very sensitive to infarct especially very early after stroke onset where it proves to be superior compared to conventional MR and CT imaging [28]. FLAIR imaging represents a T2-weighted imaging protocol in which the signal from the cerebrospinal fluid is suppressed. FLAIR images provide high sensitivity for acute and subacute infarcts [29]–[31]. For lesion delineation, we used DWI imaging within the first 48 h post-stroke and FLAIR sequences when imaging was conducted 48 h or later after stroke onset [29]–[32]. The mean time between stroke and imaging and clinical investigation for the thalamic stroke patients was 9.6 (SD 6.1, range 4–18) days in the group with pusher syndrome and 7.2 (SD 7.9, range 2–23) days in the group without the disorder (t = 0.56, p = 0.591, two-tailed). For the patients with extra-thalamic lesions the mean time was 3.5 (SD 4.7, range 0–10) days in the group of pusher patients and 3.0 (SD 4.1, range 0–9) days in the group without pusher snydrome (t = 0.16, p = 0.878, two-tailed). Scans were obtained on a 1.5-T echoplanar imaging (EPI) capable system (Magnetom Sonata, Siemens, Erlangen, Germany). The FLAIR sequence was acquired with 72 axial slices (thickness 1 mm, interslice gap 1 mm), a field of view (FOV) of a 192×256 mm^2^, matrix 192×256 pixels, repetition time (TR) of 9310 ms and an echo time (TE) of 122 ms. DWI was performed with a single-shot EPI spin echo sequence (TR 3200 ms; TE 87 ms; FOV 230×230 mm^2^; matrix 128×128 pixels; slice thickness 5 mm; gap 1mm; b-values of 0, 500 and 1000 s/mm^2^). The boundary of the lesion was delineated directly on the individual MRI image for every single transverse slice using MRIcron software [33] (http://www.mricro.com/mricron). In order to illustrate the common region of structurally lesioned brain tissue per group, both the scan and lesion shape were then transferred into stereotaxic space using the spatial normalization algorithm provided by SPM2 (http://www.fil.ion.ucl.ac.uk/spm/). For determination of the transformation parameters, cost-function masking was employed [34]. In patients with thalamic strokes, left and right lesions had been found to affect homologues structures [13], [15]. In the present analysis, we thus switched the left-sided thalamic lesions and relative perfusion maps to the right side in order to obtain a larger data basis for the subtraction analysis [35].

Hypoperfused brain tissue was visualized using perfusion-weighted imaging (PWI [36]). Fifty repetitions of perfusion-weighted EPI sequences (TR 1440 ms; TE 47 ms; FOV 230×230 mm^2^; matrix 128×128; 12 axial slices; slice thickness 5 mm; gap 1 mm) were obtained with 20 ml gadolinium diethyl triamineene pentaacetic acid (Gd-DTPA) bolus power injected at a rate of 3–5 ml/s. The amount of bolus used depended on the body-weight of the subject. Time-to-peak (TTP) maps were calculated to characterize malperfusion. TTP represents the time at which the largest signal drop occurs in the signal intensity curve with respect to the first image. It is generated directly from the signal intensity curve and does not rely on deconvoluting algorithms or the choice of adequate input functions [37], [38]. In order to identify common regions of perfusion abnormality, the PWI volumes were spatially realigned and then transferred into stereotaxic space using the spatial normalization algorithm provided by SPM2. The normalized TTP maps were spatially smoothed with a Gaussian filter of 2 mm. For SPM normalization, we used a template featuring symmetrical left-right hemispheres [39]. Subsequently voxel-wise inter-hemispheric comparisons were performed for each individual before extracting perfusion deficit volumes. This method takes regional biases for perfusion parameters into account, as each region is compared voxel-by-voxel to its mirrored region, thereby comparing homologous regions and avoiding a region-specific bias [40]. For the normalized TTP maps, we subtracted from each voxel of the affected hemisphere its mirrored voxel in the unaffected hemisphere. For the determination of volumes with perfusion abnormalities we defined the threshold for TTP delays ≥3.0 s. The TTP delay threshold was based on previous observations that TTP delays >2.5 s in Wernicke's area were associated with language dysfunction [41], and that the general functional impairment of stroke patients correlated best with the volume of PWI abnormality for TTP delays ≥4 s [42]. The area of mismatch between DWI/FLAIR and PWI abnormalities, i.e. the zones of structurally intact but dysfunctional neural tissue, was determined by subtracting for each subject the normalized DWI/FLAIR map from the normalized TTP delay map. Finally, we compared perfusion abnormalities in the patient groups with and without pusher syndrome. For this purpose, the superimposed mismatch images of the groups without pusher syndrome were subtracted from the overlap mismatch images of the groups with pusher syndrome (details concerning the subtraction technique are given in ref. [35]).

## Results

### Thalamic brain lesions

**Figure 1:**
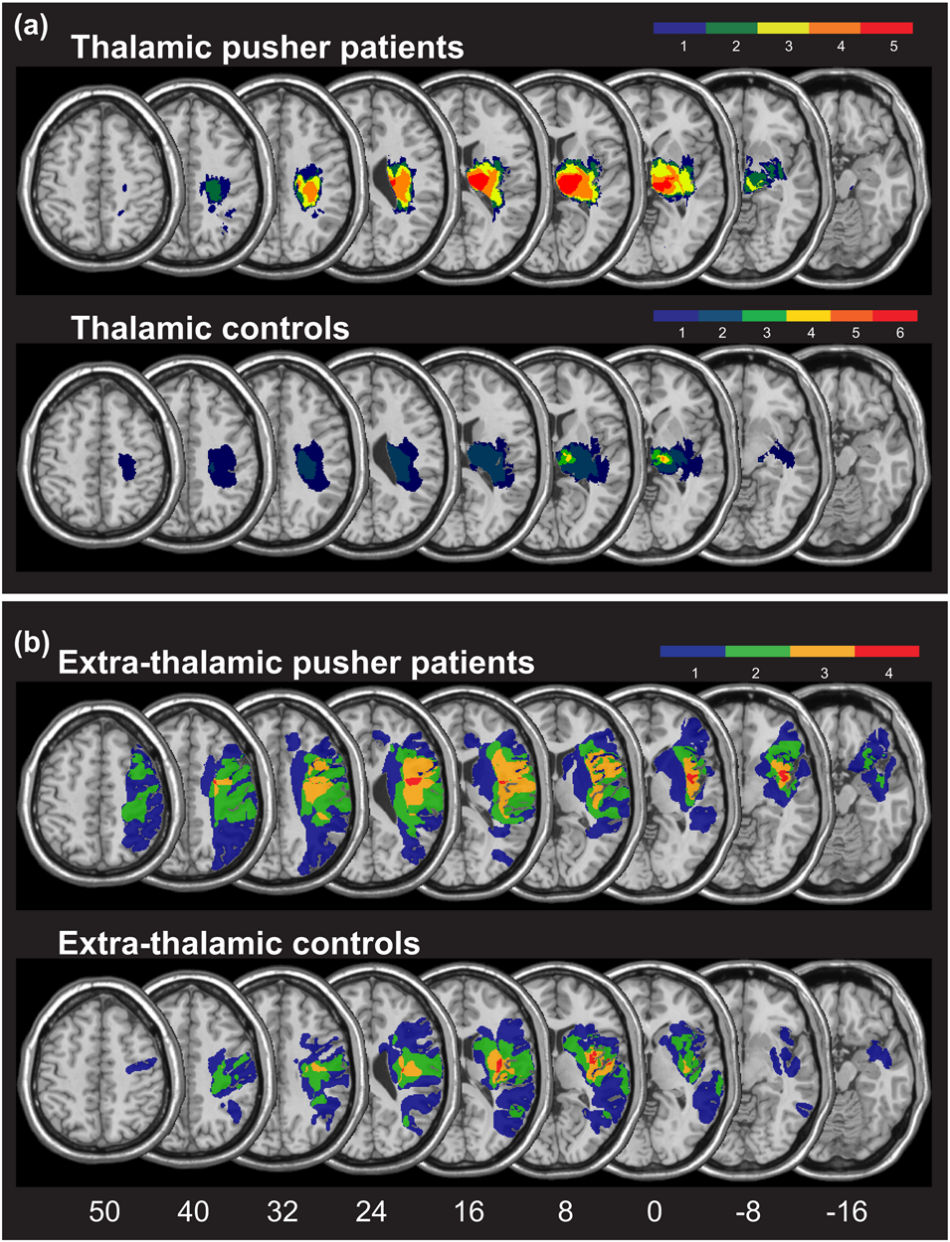
Structural lesions of all patient groups investigated. Overlay plots of the normalised structural lesions (based on normalized DWI or FLAIR images) for the groups of patient with and without pusher syndrome after (A) thalamic lesions and (B) extra-thalamic lesions. The number of overlapping areas is illustrated by different colours, coding increasing frequencies from dark blue (n = 1) to red (n = max). MNI z-coordinates of the transverse sections are given.

**Figure 2:**
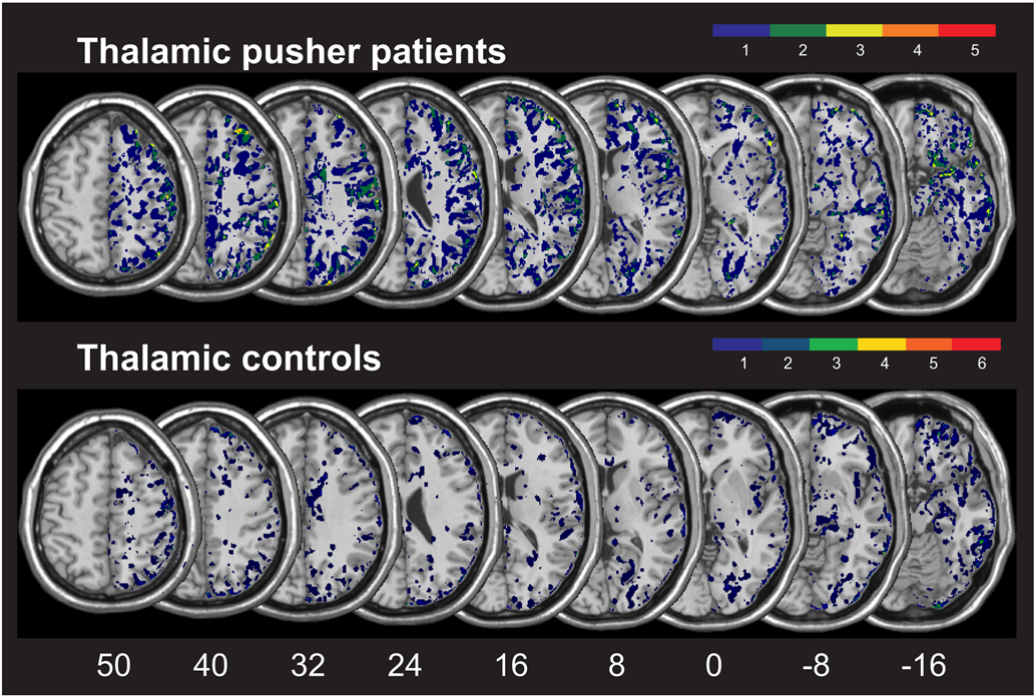
Malperfusion of structurally intact brain areas in the patient groups with thalamic lesions. Overlay plots of the patient groups with thalamic lesions showing vs. not showing pusher syndrome. Illustrated are the common regions of structurally intact but malperfused brain tissue, i.e. the mismatch between TTP abnormalities and DWI/FLAIR. The number of overlapping areas are illustrated by different colours coding increasing frequencies from dark blue (n = 1) to red (n = max). MNI z-coordinates of the transverse sections are given.

Figure 1a presents the overlay plots of the normalized DWI/FLAIR data for the group of patients with thalamic lesions showing versus not showing pusher syndrome. In both groups, the maximum of lesion overlap centered on the thalamus. In order to identify those areas that were structurally intact but hypoperfused, i.e. the zones showing a mismatch between DWI/FLAIR and PWI abnormalities, we subtracted the normalized DWI/FLAIR map from the normalized TTP delay map for each subject. The zones of perfusion abnormalities then were superimposed creating an normalized overlap image showing the common regions of structurally intact (no DWI/FLAIR abnormalities) but abnormally perfused tissue in each group (Fig. 2). In the group of patients with as well as without pusher syndrome, we found only few voxels in single patients (dark blue colour in Fig. 2 indicates malperfusion in n = 1 subject) to be malperfused though structurally intact. We analysed a further marker for abnormal perfusion, namely the maximal signal reduction (MSR). While TTP is a parameter that depicts the arrival time of blood in the brain tissue, MSR measures the amount of blood flow reaching the different regions of the brain and is closely related to relative cerebral blood flow (rCBF) in stroke patients [43], [44]. However, also the analysis of malperfused tissue as depicted by normalised MSR maps did not reveal significant perfusion changes outside the area of structural damage. Thus, we conclude that the patients with pusher syndrome following thalamic lesions did not show a systematic involvement of dysfunctional brain areas in addition to the ones found to be structurally damaged.

### Extra-thalamic brain lesions

Figure 1b presents the overlay plots of the normalized DWI/FLAIR data for the group of patients with extra-thalamic lesions showing versus not showing pusher syndrome. By using the anatomical parcellation of the MNI single-subject brain by Tzourio-Mazoyer et al. [45] implemented in MRIcron software [33] (http://www.mricro.com/mricron) and the Jülich probabilistic cytoarchitectonic atlas for the white matter fiber tracts [46], [47], we found the center of lesion overlap for the patients with pusher syndrome affecting the insula, frontal and rolandic operculum, inferior frontal gyrus, pre- and postcentral gyri, as well as part of the corticospinal tract, inferior occipitofrontal and uncinate fasciculi. The lesions of the extra-thalamic group without pusher syndrome centered on the insula, rolandic operculum, superior temporal gyrus as well as part of the corticospinal tract.

**Figure 3:**
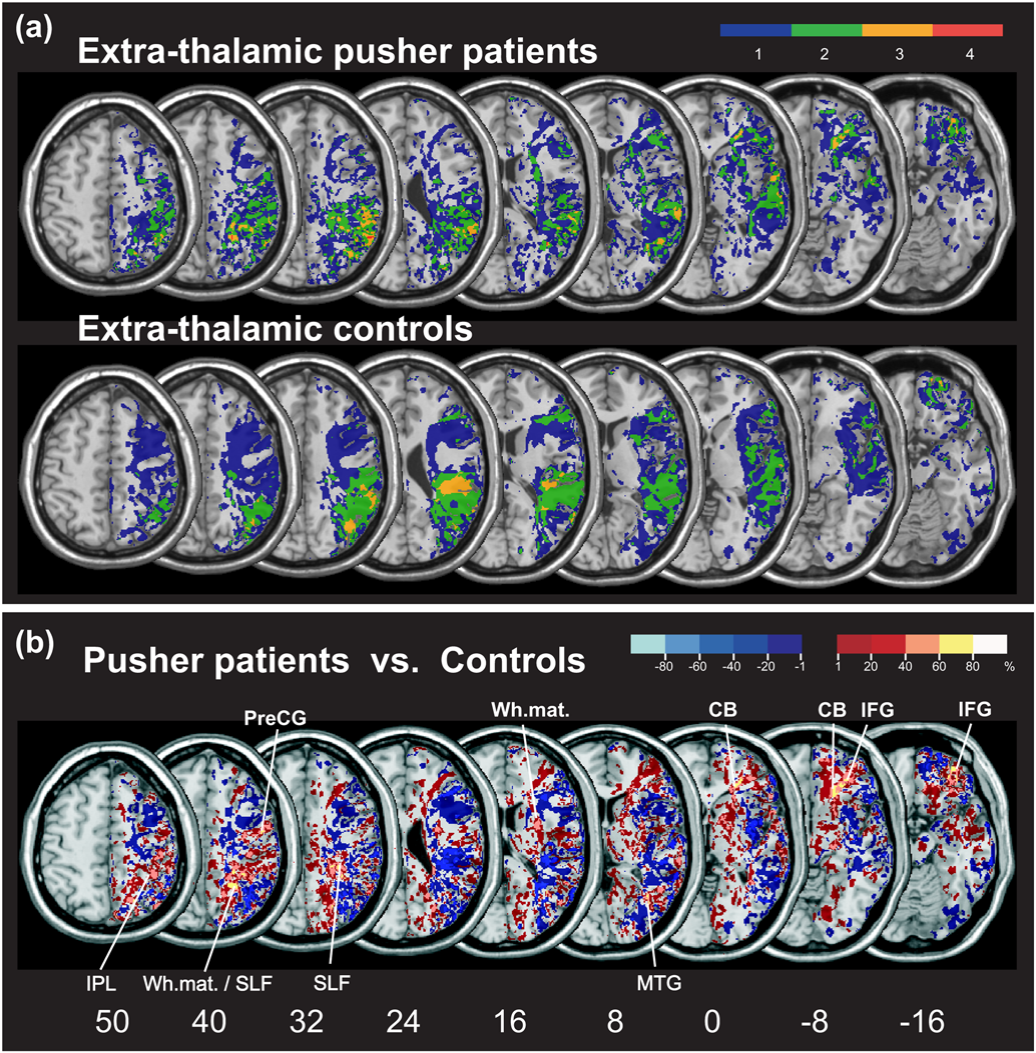
Malperfusion of structurally intact brain areas in the patient groups with extra-thalamic lesions. (A) Overlay plots of the normalised TTP delay maps showing the common regions of mismatch between DWI/FLAIR and PWI abnormalities, i.e. of structurally intact but abnormally perfused tissue, for the groups of patients with extra-thalamic lesions showing as well as not showing pusher syndrome. The number of overlapping areas with abnormal perfusion is illustrated by different colours, coding increasing frequencies from dark blue (n = 1) to red (n = max.). (B) Overlay plot of the subtracted superimposed mismatch images of the pusher group minus the mismatch images of the group without pusher syndrome. The percentage of overlapping areas of structurally intact but abnormally perfused tissue in the pusher group after subtraction is illustrated by five different colours, coding increasing frequencies from dark red (difference = 1–20%) to white (difference = 81–100%). Each colour represents 20% increments. The different colours from dark blue (difference = −1% to −20%) to light blue (difference = −81% to −100%) indicate regions abnormally perfused more frequently in patients without pusher syndrome than in the pusher group. Regions where there is an identical percentage of abnormal perfusion in both groups ( = 0%) are not depicted in the figure. MNI z-coordinates of the transverse sections are given. IFG, inferior frontal cortex; PreCG, precentral gyrus; SLF, superior longitudinal fasciculum; MTG, middle temporal cortex; CB, callosal body; Wh.mat., white matter; IPL, inferior parietal lobule.

In order to identify those areas that were structurally intact but hypoperfused, i.e. the zones showing a mismatch between DWI/FLAIR and PWI abnormalities, we subtracted the normalized DWI/FLAIR map from the normalized TTP delay map for each subject. The mismatch images of all individuals in the group of patients with pusher syndrome and in the group without the disorder were superimposed (Fig. 3a). To illustrate the common area of malperfusion in the patients with pusher syndrome in direct contrast to those malperfused areas that were present in the patients without the disorder, we subtracted the overlay mismatch images of the latter group from the overlap mismatch images of the pusher patient group. The resulting subtraction images specifically highlight structurally intact regions that were both typically hypoperfused in patients with pusher syndrome as well as typically spared in patients without the disorder (Fig. 3b). In the patients with pusher syndrome, we found the maximum of perfusion deficits in the structurally intact inferior frontal gyrus from MNI coordinates (x, 35; y, 38; z, −16) over (x, 33; y, 40; z, −8) to (x, 39; y, 41; z, 0), the middle temporal gyrus (x, 40; y, −64; z, 8), precentral gyrus (x, 51; y, −5; z, 40), inferior parietal lobule (x, 40; y, −42; z, 50), and parietal white matter at coordinates (x, 24; y, −52; z, 40). Further, small parts of the callosal body from coordinates (x, 22; y, 29; z, −8) to (x, 27; y, 36; z, 0), of the temporal white matter (x, 30; y, 1; z, 16), and of the superior longitudinal fasciculus from (x, 31; y, −37; z, 32) to (x, 26; y, −40; z, 40) were affected.

## Discussion

We examined the functioning of the structurally intact cortical tissue in patients with thalamic and with extra-thalamic strokes showing versus not showing pusher syndrome in a continuous series of stroke patients admitted to the Center of Neurology. In the patients with pusher syndrome following thalamic lesions, we found no systematic involvement of dysfunctional brain areas in addition to the ones observed to be structurally damaged. Obviously, additional cortical malperfusion is not an indispensable prerequisite if thalamic patients exhibit pusher syndrome. However, due to the limited number of 11 patients with thalamic lesions that could be investigated in the present study, we cannot exclude the possibility that a thalamic lesion combined with a perfusion deficit extending the borders of the lesion territory may also be observed in association with pusher syndrome. Our results only demonstrate that this obviously is not a physiological necessity when patients with structural damage of the thalamus exhibit pusher syndrome.

In the group of patients with extra-thalamic lesions and pusher syndrome, the thalamus was neither structurally damaged nor malperfused. Rather, these patients showed small regions of abnormal perfusion in the structurally intact inferior frontal gyrus (IFG), middle temporal gyrus (MTG), precentral gyrus, inferior parietal lobule (IPL), and parietal white matter. Further, small parts of the callosal body, of the temporal white matter, and of the superior longitudinal fasciculus (SLF) were more frequently involved. These anatomically intact but malperfused structures thus appear to contribute to the appraisal of disturbed postural control in pusher syndrome following extra-thalamic lesions.

While we have to consider, of course, the general restrictions related to the present methodology by using perfusion-weighted MR imaging, our results may indicate on the one hand that the neural tissue in the posterior thalamus itself rather than additional malperfusion in distant cortical areas is integral to perceiving gravity and controlling upright body orientation. However, the analysis also showed that thalamic damage is not a *conditio sine qua non* for the manifestation of pusher syndrome. In patients showing this disorder after extra-thalamic lesions, the thalamus was neither structurally damaged nor malperfused. This indicates that the malperfused areas uncovered in the present study as well as the structural damage in extra-thalamic areas identified in an earlier study, i.e. the insula, superior temporal gyrus, postcentral gyrus, and inferior parietal lobule [16], contribute to the network controlling upright body posture.

Interestingly, invasive studies in non-human primates [48]–[50] and *in vivo* studies in humans [51], [52] showed that some of the cortical structures lesioned in pusher syndrome, namely the insular cortex and the postcentral gyrus [16], have direct connections with the ventroposterior and the lateral posterior nuclei of the posterior thalamus, i.e. with those thalamic strucutres typically found affected when patients exhibit pushing behaviour [13], [15]. In detail, the axons arising in the ventroposterolateral and the ventroposteromedial nuclei project to primary somatosensory cortex in the postcentral gyrus (Brodmann areas 3a, 3b, 1, and 2), to the secondary somatosensory cortex in the parietal operculcum, and to the insula [49], [53]. Hence, these thalamic and cortical structures that cause pusher syndrome when lesioned might represent those areas in which the afferent sensory graviceptional signals, required to control upright body position, are processed. This conclusion is strengthen by functional imaging data that argued for a functional connectivity between these areas. For example, stimulation of the vagus nerve influenced neural activity in the thalamus, the insular cortex and the postcentral gyrus, among other brain areas [54]–[56].

The insula, the inferior parietal lobule, the superior temporal gyrus and the postcentral gyrus, have also been recognized to be the substrate of visuo-vestibular processing [8], [20], [57]–[60]. Unilateral lesions of the superior temporal and of the insular cortices (including the parieto-insular vestibular cortex [PIVC]) cause deviations of the perceived subjective visual vertical (SVV) and lateral imbalance of stance and gait [4], [20], [59], [61]. The question thus raises whether those brain areas representing the visual-vestibular system might also be related to the control of upright body orientation studied in the present experiment.

Our study does not allow to answer this question. At the behavioural level, these two processes clearly dissociate. While patients with vestibular disorders have abnormal tilts of the SVV but preserved perception of postural vertical [3], pusher patients show the opposite patter, namely a preserved perception of the SVV with a marked tilted perception of own body posture [9], [11]. It remains the issue of future studies to investigate whether these two behavioural processes are mediated by anatomically identical or closely related cortical structures in the IPL, STG and postcentral gyrus.

To conclude, the present research supports the assumption that malfunction or lesion of cortical areas not involving the thalamus can indeed be associated with pushing behaviour. Vice versa the data suggest that if the posterior thalamus is lesioned in pusher patients it is the damage of the neural tissue in the posterior thalamus itself, and not necessarily malperfusion in distant cortical areas, that provokes the behavioural disorder. Thus, it seems as if the normal functioning of both extra-thalamic as well as posterior thalamic structures is integral to perceiving gravity and controlling upright body orientation in humans.
